# The Effect of Surgeon Expertise on the Outcome of Patients with Adrenocortical Carcinoma

**DOI:** 10.3390/jpm12010100

**Published:** 2022-01-13

**Authors:** Anja Barac Nekic, Nikola Knezevic, Karin Zibar Tomsic, Ivana Kraljevic, Annemarie Balasko, Tanja Skoric Polovina, Mirsala Solak, Tina Dusek, Darko Kastelan, Croatian ACC Study Group

**Affiliations:** 1 Department of Internal Medicine, General Hospital Dubrovnik, Roka Misetica 2, 20000 Dubrovnik, Croatia; 2 Department of Urology, University Hospital Centre Zagreb, Kispaticeva 12, 10000 Zagreb, Croatia; nknezevic5@yahoo.com; 3 School of Medicine, University of Zagreb, Salata 2, 10000 Zagreb, Croatia; ivana.kraljevic@gmail.com (I.K.); tina.dusek@gmail.com (T.D.); darko.kastelan@gmail.com (D.K.); 4 Department of Endocrinology, University Hospital Center Zagreb, Kispaticeva 12, 10000 Zagreb, Croatia; karinzibar@gmail.com (K.Z.T.); anakegalj@gmail.com (A.B.); skoric.polovina@gmail.com (T.S.P.); mirsala.solak@gmail.com (M.S.)

**Keywords:** adrenocortical carcinoma, adrenal surgery, patient outcome

## Abstract

Complete surgical removal of adrenocortical carcinoma (ACC) represents the only chance of long-term cure. In this study, we compared the long-term outcomes of ACC patients depending on whether they had adrenal surgery performed in a high-volume (HVC) or in a low-volume (LVC) center. This retrospective study included 49 patients from the Croatian ACC Registry with the European Network for the Study of Adrenal Tumors (ENSAT) stage I–III ACC, of which 35 underwent surgery in a HVC whereas 14 of them were operated in one of the LVCs. Patients operated in the LVCs had a significantly higher rate of ACC recurrence (57.1% vs. 22.9%; *p* = 0.02). Accordingly, RFS was significantly longer in patients operated on in HVC (*p* = 0.04). The difference in RFS remained significant after controlling for age, gender, tumor size, Ki-67 index, Weiss score, and type of surgery (HR 4.55; 95% CI 1.16–17.88; *p* = 0.03). In addition, there is a tendency towards longer DSS in patients in the HVC group compared to those in the LVC group (*p* = 0.05). These results point to the centralization of adrenal surgery as a key prerequisite for improving the outcomes of ACC patients.

## 1. Introduction

Management of rare diseases poses a major challenge to health systems in many ways. Patients with a rare disease commonly face the problem of a lack of specialized and coordinated medical care, resulting in a poorer outcome of their treatment. In this regard, centralized management of such patients is essential to improve the quality of their health care. Adrenocortical carcinoma (ACC) is a rare and aggressive neoplasm in which complete surgical removal of the tumor represents the only chance of a long-term cure [1]. Accordingly, access to a surgeon with expertise in adrenal and oncological surgery is of the utmost importance to optimize patients’ outcome [2,3,4]. However, a few recent studies reported that a large number of adrenal surgeons perform a small number of adrenalectomies per year [5,6]. Moreover, these studies demonstrated that patients managed by low-volume surgeons were more likely to experience postoperative complications, underscoring the need for a more centralized healthcare service for adrenal tumors. The most recent guidelines recommend ACC surgery to be performed in a high-volume center by a surgeon with expertise in both open and laparoscopic adrenal surgery [7]. This was supported by several studies showing that patients with ACC operated on in high volume centers had better prognosis in terms of lower recurrence rate and improved survival [8,9,10]. 

However, although several studies reported the results of adrenal surgery in patients with ACC, a relatively small number of them compared patients’ outcomes depending on center volume and surgeon expertise. Furthermore, the retrospective nature of these studies, with results potentially affected by important confounding factors, makes it difficult to draw definite conclusions. To add to the existing knowledge, this study compared the long-term outcomes of ACC patients depending on whether they had adrenal surgery performed in a high-volume (HVC) or in a low-volume (LVC) center.

## 2. Materials and Methods

In this retrospective study, we included patients from the Croatian ACC Registry with the European Network for the Study of Adrenal Tumors (ENSAT) stage I–III ACC who had adrenal surgery between 2008 and 2020. Of 49 eligible patients, 35 underwent surgery in a HVC by a single surgeon, whereas 14 of them were operated on in one of the LVCs. Data on demographics, hormonal status, imaging, surgical and pathology reports, adjuvant treatment, and follow-up were collected from hospital records. The study was approved by the University Hospital Centre Zagreb Ethics committee. 

The diagnosis of ACC was based on Weiss histopathological criteria, except in patients with oncocytic ACC, in whom Lin–Weiss–Bisceglia scoring was used [7,11]. Tumor stage was determined using the ENSAT classification system [7]. 

The centers were considered HVC if they had an average of >20 adrenal surgeries per year per surgeon in the last 10 years, of which at least two patients per year had ACC. All patients in the HVC were operated on by a single surgeon who was a urologist, whereas in the LVC, patients were operated on by a urologist or abdominal surgeon.

The main outcomes of the study were differences between the groups in recurrence-free survival (RFS) and disease specific survival (DSS). RFS was calculated from the date of the ACC surgery to the date of tumor recurrence or the last imaging follow-up. DSS was calculated from the date of ACC surgery to the date of death related to ACC or the last follow-up visit. 

### Statistical Analysis

Statistical analysis was performed using SPSS version 17.0 for Windows. Variables were described as median (minimum-maximum) and as numbers (percentages). The difference between two independent numerical variables was tested using the Mann–Whitney test, and the χ^2^ test was applied for testing the difference between two categorical variables. Survival analysis was performed using the Kaplan–Meier method and differences were assessed with the log-rank test. For multivariate analysis, the Cox regression model was applied. A confidence interval of 95% was used. The statistical significance level was set at *p* < 0.05.

## 3. Results

### 3.1. Patient Characteristics

In this retrospective study, we included 49 patients with ACC, ENSAT stage I–III, of whom 35 were operated on in the HVC, whereas 14 patients underwent surgery in one of the seven LVCs. There were no differences between the groups regarding age, gender, ENSAT tumor stage, excess glucocorticoid secretion, Weiss score, Ki-67 index, adjuvant mitotane treatment, and duration of follow up. There was a trend to a larger tumor size in the LVC group (80 (26–176) mm vs. 107.5 (70–250) mm; *p* = 0.05). The laparoscopic method was more frequently used in the HVC group compared to the LVC group (71.4% vs. 28.6% of patients; *p* = 0.006). Twenty-one patients in the HVC group and six in the LVC group received adjuvant mitotane treatment. The patient characteristics are shown in Table 1.

### 3.2. Long-Term Outcomes

Disease recurrence was observed in 16 patients, eight in each group (HVC 22.9% vs. LVC 57.1%; *p* = 0.02). Eleven patients had local tumor recurrence or peritoneal carcinomatosis, with four (11.4%) in the HVC group and seven (50%) in the LVC group (*p* = 0.02). Furthermore, three patients in the LVC group, of which one had an intraoperative tumor spillage, and none in the HVC group had disease recurrence within six months after the surgery. No difference in recurrence rate was observed between the open and laparoscopic approach (*p* = 0.157). 

**Figure 1:**
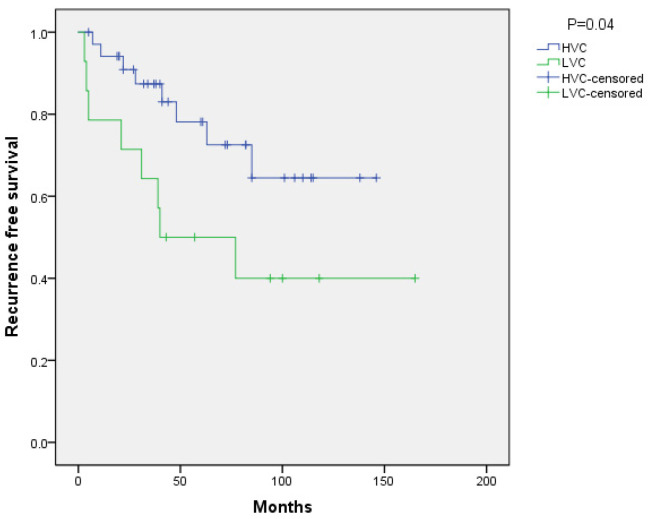
Recurrence free survival.

Patients in the HVC group had significantly longer RFS than those in the LVC group (*p* = 0.04; Figure 1) and the difference remained significant after controlling for age, gender, tumor size, Ki-67 index, Weiss score, and type of surgery (HR 4.55; 95% CI 1.16–17.88; *p* = 0.03). 

**Figure 2:**
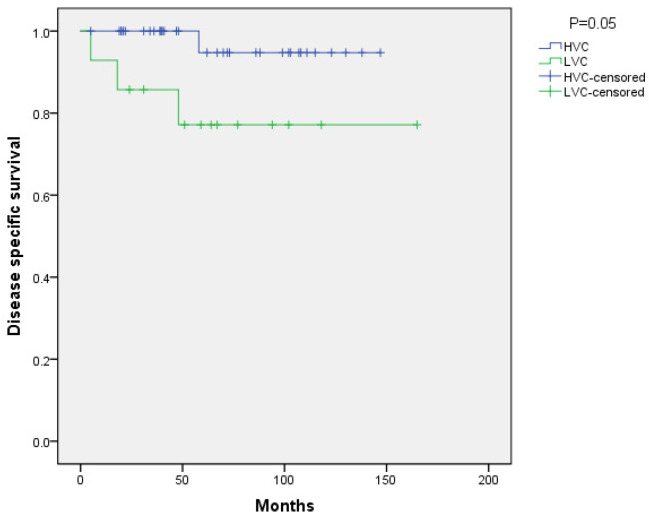
Disease specific survival.

In terms of survival, eight patients died during follow-up (4 HVC and 4 LVC), and in four of them (1 HVC and 3 LVC), the death was related to ACC (*p* = 0.03). There was no difference between the groups in overall survival. However, there is a trend to better DSS in patients in the HVC group compared to those in the LVC group (*p* = 0.05; Figure 2). 

## 4. Discussion

Adrenal surgery is the cornerstone of ACC treatment and provides the best chance for disease cure. To ensure an optimal outcome, it is of greatest importance that the surgery is performed by a surgeon with expertise in both adrenal and oncologic surgery. However, literature data showed that many adrenal surgeries are done by surgeons who perform only a few such operations per year [12]. Experiences from other areas of oncology show that a lower surgeon expertise has a negative impact on the patients’ outcome [13,14,15]. Likewise, our study demonstrated lower ACC recurrence rate and longer RFS in patients who were operated on by an expert surgeon. In addition, a trend toward improved survival rate has also been observed. A few other studies that explored the effect of hospital volume on ACC patient’s outcome obtained similar results. Kerkhofs et al. analyzed the outcomes of 189 patients with local or locally advanced ACC, and they demonstrated improved survival in patients operated on in hospitals that were part of the Dutch Adrenal Network [9]. Furthermore, the Italian multicentric surgical survey with 263 patients reported that those who underwent adrenalectomy for ACC in ‘high-volume’ centers had better oncologic outcomes in terms of a lower recurrence rate and longer time to recurrence [4]. Finally, a study by Grubbs et al. indicated that surgery in a non-specialized institution was associated with an increased risk of disease recurrence [16].

Overall, surgeon experience has an impact predominantly on the risk of local recurrence and spreading of cancer cells inside the abdominal cavity. In our cohort, four patients (11.4%) operated on in the HVC had local recurrence, whereas none of the patients had peritoneal carcinomatosis. In contrast, in the LVC group, local recurrence and peritoneal carcinomatosis were observed in six and in one patient, respectively (50%; *p* = 0.02). Furthermore, in three patients from the LVC group and in none from the HVC group, ACC recurrence was noted already within six months after the surgery. 

In addition to surgeon expertise, other clinical and biochemical factors might influence the risk of ACC recurrence [1,17]. In this regard, no differences were observed between the HVC and LVC groups in age, gender, tumor ENSAT stage, Ki-67 index, Weiss score, excess glucocorticoid secretion, and adjuvant mitotane treatment. However, there is a trend to larger tumors in patients in the LVC group (*p* = 0.05). Regarding the type of surgery, laparoscopic adrenalectomy was used more frequently in the HVC group (*p* = 0.005). In a multivariate analysis, after controlling for age, gender, tumor size, Ki-67 index, Weiss score, and type of surgery, the difference between the groups in RFS remained significant. 

Various factors may contribute to a more favorable ACC treatment outcome in expert centers. Center volume and surgical experience are crucial in this regard [18,19], but other factors, such as optimal post-surgical care, including a standardized follow-up protocol by a multidisciplinary team of experts and adjuvant mitotane treatment, could also play an important role. In the present study, the majority of the patients operated in the LVCs were referred to the HVC within three months after the surgery, and they were subjected to identical follow-up monitoring and treatment protocol as the patients operated on in the HVC. However, two patients from the LVC group were referred to an expert center more than three months after surgery when they already had a relapse of disease. 

The retrospective nature and a small number of patients are major shortcomings of the study. Furthermore, the study results might be affected by selection bias, as there is a possibility that a certain proportion of patients who were operated on in the LVCs and had a low risk of disease recurrence were not referred to the HVC for further management. However, given the ACC incidence of 0.5–2 cases per million people, we believe that our group of 49 patients almost represents the entire cohort of Croatian patients with ACC ENSAT stage I–III. Another limitation refers to the criteria we used to define adrenal surgery HVC. Although there is no consensus on that subject, the recent ESMO-EURACAN clinical guidelines suggest that centers having >20 adrenal surgeries per year meet the criteria for HVC [20]. However, in addition to expertise in adrenal surgery, experience in oncological surgery is also essential for the optimal surgical management of ACC. Therefore, in the present study, an HVC was defined with >20 adrenal surgeries per year in the last five years, of which at least two per year concern ACC. In conclusion, the results of our study showed that ACC surgery performed in expert centers was associated with a better oncological outcome in terms of lower risk of disease recurrence and a tendency towards improved survival rate. These findings point to the centralization of adrenal surgery as a key prerequisite for improving outcomes of ACC patients. Finally, the complexity of medical care of these patients requires multidisciplinary input and, therefore, management of ACC patients should be restricted to institutions in which such input can be provided. 

## Figures and Tables

**Table 1:** Patient demographics and clinical characteristics.

Variable	HVC (*n* = 35)	LVC (*n* = 14)	*p*-Value
Age (year)	46 (18–77)	56.5 (24–78)	0.22
Female gender (n, %)	24 (68.5)	10 (71.4)	0.85
Tumor size (mm)	80 (26–176)	107.5 (70–250)	0.05
ENSAT tumor stage (n, %)			
Stage I	6 (17.1)	0(0)	
Stage II	21 (60.0)	10 (71.4)	
Stage III	8 (22.9)	4(28.6)	
Excess glucocorticoid secretion (*n*, %)	13 (37.1)	4 (28.6)	0.57
Ki-67 (%)	12 (1–60)	14 (4–65)	0.25
Weiss score	6 (3–9)	6.5 (4–8)	0.79
Adjuvant mitotane (*n*, %)	21 (60)	6 (42.9)	0.28
Follow-up (months)	62 (5–147)	61.5(5–165)	0.85
Laparoscopic surgery (*n*, %)	25 (71.4)	4(28.6)	0.006
Recurrence (*n*, %)	8 (22.9)	8(57.1)	0.02
Laparoscopic surgery (*n*, %)	6(24)	3(75)	0.157
Open surgery (*n*, %)	2(20)	5(50)	
Death (*n*, %)	4(11.4)	4(28.6)	0.14

High-volume (HVC), Low-volume (LVC).

## Data Availability

The data presented in this study are available on request from the corresponding author. The data are not publicly available due to privacy reasons.

## References

[1] Kastelan D., Muzurovic E., Dusek T. (2021). Approach to Patients with European Network for the Study of Adrenal Tumor Stages I and II Adrenocortical Carcinomas. Curr. Opin. Endocrinol. Diabetes Obes..

[2] Stavrakis A.I., Ituarte P.H.G., Ko C.Y., Yeh M.W. (2007). Surgeon Volume as a Predictor of Outcomes in Inpatient and Outpatient Endocrine Surgery. Surgery.

[3] Villar J.M., Moreno P., Ortega J., Bollo E., Ramírez C.P., Muñoz N., Martínez C., Domínguez-Adame E., Sancho J., Del Pino J.M. (2010). Results of Adrenal Surgery. Data of a Spanish National Survey. Langenbeck’s Arch. Surg..

[4] Lombardi C.P., Raffaelli M., Boniardi M., De Toma G., Marzano L.A., Miccoli P., Minni F., Morino M., Pelizzo M.R., Pietrabissa A. (2012). Adrenocortical Carcinoma: Effect of Hospital Volume on Patient Outcome. Langenbeck’s Arch. Surg..

[5] Palazzo F., Dickinson A., Phillips B., Sahdev A., Bliss R., Rasheed A., Krukowski Z., Newell-Price J. (2016). Adrenal Surgery in England: Better Outcomes in High-Volume Practices. Clin. Endocrinol..

[6] Al-Qurayshi Z., Robins R., Buell J., Kandil E. (2016). Surgeon Volume Impact on Outcomes and Cost of Adrenal Surgeries. Eur. J. Surg. Oncol..

[7] Fassnacht M., Dekkers O.M., Else T., Baudin E., Berruti A., De Krijger R.R., Haak H.R., Mihai R., Assie G., Terzolo M. (2018). European Society of Endocrinology Clinical Practice Guidelines on the Management of Adrenocortical Carcinoma in Adults, in Collaboration with the European Network for the Study of Adrenal Tumors. Eur. J. Endocrinol..

[8] Fassnacht M., Johanssen S., Fenske W., Weismann D., Agha A., Beuschlein F., Führer D., Jurowich C., Quinkler M., Petersenn S. (2010). Improved Survival in Patients with Stage II Adrenocortical Carcinoma Followed up Prospectively by Specialized Centers. J. Clin. Endocrinol. Metab..

[9] Kerkhofs T.M.A., Verhoeven R.H.A., Bonjer H.J., Nieveen Van Dijkum E.J., Vriens M.R., De Vries J., Van Eijck C.H., Bonsing B.A., Van De Poll-Franse L.V. (2013). Surgery for Adrenocortical Carcinoma in the Netherlands: Analysis of the National Cancer Registry Data. Eur. J. Endocrinol..

[10] Gonzalez R.J., Shapiro S., Sarlis N., Vassilopoulou-Sellin R., Perrier N.D., Evans D.B., Lee J.E. (2005). Laparoscopic Resection of Adrenal Cortical Carcinoma: A Cautionary Note. Surgery.

[11] Bisceglia M., Ludovico O., Di Mattia A., Ben-Dor D., Sandbank J., Pasquinelli G., Lau S.K., Weiss L.M. (2004). Adrenocortical Oncocytic Tumors: Report of 10 Cases and Review of the Literature. Int. J. Surg. Pathol..

[12] Mihai R., Donatini G., Vidal O., Brunaud L. (2019). Volume-Outcome Correlation in Adrenal Surgery-an ESES Consensus Statement. Langenbeck’s Arch. Surg..

[13] Van De Poll-Franse L.V., Lemmens V.E.P.P., Roukema J.A., Coebergh J.W.W., Nieuwenhuijzen G.A.P. (2011). Impact of Concentration of Oesophageal and Gastric Cardia Cancer Surgery on Long-Term Population-Based Survival. Br. J. Surg..

[14] Lemmens V.E.P.P., Bosscha K., van der Schelling G., Brenninkmeijer S., Coebergh J.W.W., de Hingh I.H.J.T. (2011). Improving Outcome for Patients with Pancreatic Cancer through Centralization. Br. J. Surg..

[15] Park H.S., Roman S.A., Sosa J.A. (2009). Outcomes from 3144 Adrenalectomies in the United States: Which Matters More, Surgeon Volume or Specialty?. Arch. Surg..

[16] Grubbs E.G., Callender G.G., Xing Y., Perrier N.D., Evans D.B., Phan A.T., Lee J.E. (2010). Recurrence of Adrenal Cortical Carcinoma Following Resection: Surgery Alone Can Achieve Results Equal to Surgery plus Mitotane. Ann. Surg. Oncol..

[17] Solak M., Kraljević I., Zibar Tomšić K., Kaštelan M., Kakarigi L., Kaštelan D. (2021). Neutrophil-Lymphocyte Ratio as a Prognostic Marker in Adrenocortical Carcinoma. Endocr. Res..

[18] Kastelan D., Knezevic N., Zibar Tomsic K., Alduk A.M., Kakarigi L., Kastelan M., Coric M., Skoric-Polovina T., Solak M., Kraljevic I. (2020). Open vs Laparoscopic Adrenalectomy for Localized Adrenocortical Carcinoma. Clin. Endocrinol..

[19] Kulis T., Knezevic N., Pekez M., Kastelan D., Grkovic M., Kastelan Z. (2012). Laparoscopic Adrenalectomy: Lessons Learned from 306 Cases. J. Laparoendosc. Adv. Surg. Tech..

[20] Fassnacht M., Assie G., Baudin E., Eisenhofer G., de la Fouchardiere C., Haak H.R., de Krijger R., Porpiglia F., Terzolo M., Berruti A. (2020). Adrenocortical Carcinomas and Malignant Phaeochromocytomas: ESMO-EURACAN Clinical Practice Guidelines for Diagnosis, Treatment and Follow-Up. Ann. Oncol..

